# Infections after high-voltage cardiac implantable electronic device replacements

**DOI:** 10.1016/j.hroo.2026.01.033

**Published:** 2026-02-18

**Authors:** Rasmus Langhoff, Amar Taha, Peter Raivio, Juha Suhonen, Pekka Raatikainen, Jarkko Karvonen, Andreas Martinsson, Markus Sane

**Affiliations:** 1 Helsinki University, Helsinki, Finland; 2 Department of Molecular and Clinical Medicine, Institute of Medicine, Sahlgrenska Academy, University of Gothenburg, Gothenburg, Sweden; 3 Region Västra Götaland, Department of Cardiology, Sahlgrenska University Hospital, Gothenburg, Sweden; 4 Helsinki University Hospital Heart and Lung Center, Helsinki, Finland

**Keywords:** Infection, CIED, Prevention, Incidence, Antibiotic envelope, ICD, CRT-D

## Abstract

**Background:**

Device infection is the most worrisome complication after replacement of cardiac implantable electronic devices, with the utmost risk among patients with replacement of a high-voltage device.

**Objective:**

This study sought to examine periprocedural approaches and investigate the contemporary infection rates after replacement of an implantable cardioverter-defibrillator or cardiac resynchronization therapy defibrillator device in 2 large university hospitals.

**Methods:**

All transvenous implantable cardioverter-defibrillator and cardiac resynchronization therapy defibrillator replacements (n = 298) performed in 2021–2023 at Helsinki University Hospital and Sahlgrenska University Hospital were included in this retrospective analysis. Baseline, clinical, and procedural data were extracted from electronic medical records, and the infection risk was assessed using the Prevention of Arrhythmia Device Infection Trial score.

**Results:**

During a mean follow-up of 24.6 ± 10.4 months after generator replacement, 3 procedure-related infections were documented, corresponding to an infection rate of 1.0%. All patients received procedural antibiotic prophylaxis, and an antibacterial envelope was used in 51.3% of the patients. There was no difference in the infection rate between the centers (0.95% vs 1.15%), although the use of an antimicrobial envelope was more common at Helsinki University Hospital than Sahlgrenska University Hospital (84.8% vs 44.2%) among high-risk patients (Prevention of Arrhythmia Device Infection Trial score ≥6).

**Conclusion:**

The incidence of procedure-related infections after high-voltage cardiac implantable electronic device replacement can be maintained at a low level when guideline-based infection prevention measures are rigorously applied.

**Key Findings**

▪ Overall, 3 procedure-related infections were documented, corresponding to an infection rate of 1.0% in a cohort of patients undergoing high-power generator replacement.
▪ The use of an antibiotic envelope varied depending on whether it was systematically applied to all patients considered high risk based on the Prevention of Arrhythmia Device Infection Trial score or left to the operator’s discretion.
▪ Applying the antibiotic envelope based on operator discretion may result in a more random use of the therapy rather than targeting individuals at the highest risk.
▪ The incidence of procedure-related infections after high-voltage cardiac implantable electronic device replacement can be kept at a low level when guideline-based infection prevention measures are rigorously implemented.

## Introduction

Infections of cardiac implantable electronic devices (CIEDs) are associated with substantial morbidity, mortality, and health care cost.^1^^,^^2^ In studies published between 2006 and 2023, infection rates after CIED generator replacements or upgrade procedures ranged from 1.0% to 5.0%,^3^, ^4^, ^5^, ^6^, ^7^, ^8^, ^9^, ^10^, ^11^, ^12^, ^13^, ^14^ and epidemiologic data suggest that the incidence of device infections has grown in recent years.^15^ The infection risk is particularly high in replacement or revision of high-voltage devices including implantable cardioverter-defibrillators (ICDs) and cardiac resynchronization therapy defibrillators (CRT-Ds).

International consensus documents have addressed several aspects of CIED infections and provided recommendations on how to minimize the risk of procedure-related complications.^1^^,^^2^ Preoperative antibiotic prophylaxis is a standard of care, and the use of an antimicrobial envelope delivering sustained-release antibiotics should be considered in high-risk patients.^1^ However, a definite classification of such patients is lacking, and therefore, the optimal patient selection for this costly additional care may be challenging.

This study aimed to evaluate the contemporary infection rate after high-voltage CIED replacement and describe current practices for prevention of CIED infections (eg, antibiotic prophylaxis, antibacterial envelopes, and procedural settings) in a real-world clinical setting.

## Methods

### Study design and data collection

This retrospective study was conducted at 2 large quaternary care centers: the Heart and Lung Center at Helsinki University Hospital (HUH) in Helsinki, Finland, and the Department of Cardiology at Sahlgrenska University Hospital (SUH) in Gothenburg, Sweden. The total catchment population of the Heart and Lung Center at HUH is 2.1 million inhabitants, and 1.7 million at the Department of Cardiology of SUH.

Universal electronic patient records are used in both health care districts, enabling comprehensive retrospective follow-up of all patients. In addition, both centers are responsible for lead extraction procedures in their catchment areas, ensuring that any patient in whom lead extraction is considered is reliably identified.

**Figure 1:**
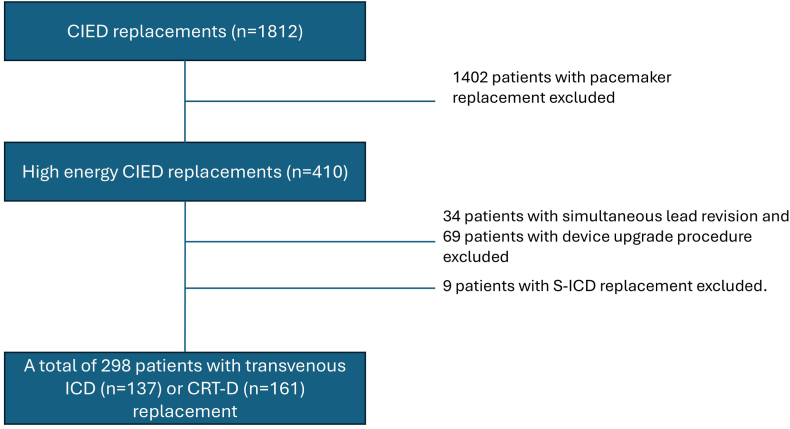
Flowchart of patient cohort. CIED = cardiac implantable electronic device; CRT-D = cardiac resynchronization therapy defibrillator; ICD = implantable cardioverter-defibrillator; S-ICD = subcutaneous implantable cardioverter-defibrillator.

All patients who underwent generator replacement of a transvenous ICD or CRT-D, between January 1, 2021, and December 31, 2023, were included (Figure 1). Patients who underwent simultaneous lead revision or device upgrade were excluded (n = 103). In addition, at HUH 1 patient was excluded owing to heart transplantation shortly after the generator replacement.

The following data were collected: patient demographics (age, sex), device-related variables (type, implantation site, pacemaker dependency), clinical variables (indication for initial implantation, underlying cardiac disease, and other comorbidities), and current medications including antiplatelet agents, anticoagulants, and immunosuppressive therapy. Procedure-related variables included the duration of the procedure, the number of previous procedures at the pocket site, and the use of an antibacterial envelope. The Prevention of Arrhythmia Device Infection Trial (PADIT) score^16^ was calculated for each patient. The score stratifies patients into low (0–4 points), intermediate (5–6 points), and high risk groups (≥7 points) based on 5 clinical factors: previous procedures (P), age (A), depressed renal function (D), immunosuppression (I), and procedure type (T). Renal insufficiency was defined as an estimated glomerular filtration rate of <30 mL/min/1.73 m^2^, consistent with the PADIT scoring system. Remote monitoring of the devices was used in all patients to enable prompt detection of battery longevity, lead and device malfunction, and tachyarrhythmias.

The incidence of infections was collected for a minimum follow-up of 6 months after the generator replacement. The primary outcome was the incidence of a procedure-related infection during the follow-up. Infections were categorized as superficial, pocket, or systemic, according to the definitions provided in the European Heart Rhythm Association consensus statement.^1^

### Preoperative and procedural workflow

Generator replacements were scheduled after an elective replacement indicator alert, typically found by remote monitoring. An overview of the preoperative examinations and perioperative workflows at HUH and SUH is presented in Table 1. Standard preoperative blood test results were reviewed before the procedure, and if necessary (eg, owing to signs of systemic infection), the procedure was postponed.

**Table 1:** Comparison of perioperative management of the generator between the 2 centers

Characteristic	HUH	SUH
Preoperative visit	Yes	Not mandatory
Preoperative blood tests	CBC, CRP, Na, K, creatinine, INR	CBC, CRP, Na, K, creatinine, INR
Antithrombotic therapy	DOAC discontinued 24–48 h	DOAC discontinued 24–36 h
	Continued VKA	Continued VKA
	ASA and ADP receptor inhibitors discontinued 5 d∗	Continued ASA and ADP receptor inhibitors
Prophylactic antibiotic	Cefuroxime 3 g IV <60 min before incision If allergic: vancomycin 1 g IV <90–120 min before incision	Cloxacillin 2 g IV <60 min before incision If allergic: clindamycin 600 mg IV <60 min before incision
Procedure location	Surgical operating room†	Catheterization laboratory
Primary operator	Cardiothoracic surgeon†	Electrophysiologist
Use of antibiotic envelope (Tyrx)	All patients with a PADIT score of ≥6	At the discretion of the operator

ADP = adenosine diphosphate; ASA = acetylsalicylic acid; CBC = complete blood count; CRP = C-reactive protein; DOAC = direct oral anticoagulant; HUH = Helsinki University Hospital; INR = international normalized ratio; IV = intravenous; K = potassium; Na = sodium; PADIT = Prevention of Arrhythmia Device Infection Trial; SUH = Sahlgrenska University Hospital; VKA = vitamin K antagonist.

∗ If the indication for therapy was a recent (<12 months) acute coronary syndrome or percutaneous coronary intervention, the treatment was continued.

† Acute cases may be performed in the catheterization laboratory by a senior electrophysiologist.

At both hospitals, the operators adhered to high surgical standards, and special care was taken to obtain meticulous hemostasis using diathermy or plasma scalpel.

### Statistical analysis

Continuous variables are reported as mean ± standard deviation or as median with interquartile range, as appropriate based on data distribution. Categorical variables are presented as counts and percentages. Owing to the small number of infections in the cohort, no inferential statistical analyses were performed, and *P* values were not reported. All statistical analyses were performed using IBM SPSS 25.0 for Windows (IBM Corp, Armonk, NY).

### Ethical aspects

This study was conducted according to the 1975 Declaration of Helsinki. Ethical approval was obtained from the Swedish Ethical Review Authority (approval number 2023-06560-01, date November 29, 2023). In Helsinki, the study was approved by the institutional review board, and after consultation with the ethics committee, it was determined that ethics approval was not required owing to the retrospective nature of the study. In addition, the review board in both institutions waived the need for consent owing to the retrospective nature of the study. The research reported in this paper adhered to the Consolidated Standards of Reporting Trials guidelines.

## Results

During the study period, a total of 298 generator replacements were performed at HUH (n = 211) and SUH (n = 87). The mean follow-up for the combined cohort was 24.6 months (± 10.5), with a mean duration of 23.3 months (± 10.4) at HUH and 27.8 months (± 10.1) at SUH. Baseline characteristics of the patients are presented in Table 2, and a summary of the procedure-related factors that may influence the risk of infection is presented in Table 3. A total of 12 patients (7 at HUH and 5 at SUH) died from causes unrelated to generator replacement during the follow-up.

**Table 2:** Baseline characteristics of the patients

Characteristic	HUH (n = 211)	SUH (n = 87)	Total (n = 298)
Age, median (IQR), y	67.9 (58.3–75.5)	71.0 (61.0–78.0)	68.8 (59.1–76.2)∗
Women, n (%)	74 (35.1)	20 (23.0)	94 (31.5)
Underlying heart diseases, n (%)			
Ischemic cardiomyopathy	64 (30.3)	41 (47.1)	105 (35.2)
Dilated cardiomyopathy	75 (35.5)	22 (25.3)	97 (32.6)
Hypertrophic cardiomyopathy	13 (6.2)	2 (2.3)	15 (5.0)
ARVC	4 (1.9)	4 (4.6)	8 (2.7)
Inflammatory cardiomyopathy	18 (8.5)	2 (2.3)	20 (6.7)
Ion channel disorder	7 (3.3)	3 (3.4)	10 (3.4)
Other	30 (14.2)	13 (14.9)	43 (14.4)
Other comorbidities, n (%)			
Diabetes mellitus	42 (35.1)	15 (17.2)	57 (19.1)
COPD	8 (3.8)	5 (5.7)	13 (4.4)
Renal insufficiency†	7 (3.3)	4 (4.6)	11 (3.7)
Heart failure	128 (60.7)	66 (75.9)	194 (65.1)
History of cancer	15 (7.1)	7 (8.0)	22 (7.4)
Active cancer	6 (2.8)	4 (4.7)	10 (3.4)
PADIT score, median (IQR)	6 (5–7)	6 (4–7)	6 (4–7)∗
Antiplatelet therapy	48 (22.7)	19 (21.8)	67 (22.5)
ASA	42 (19.9)	18 (20.7)	60 (20.1)
Clopidogrel	5 (2.4)	1 (1.1)	6 (2.0)
Dual antiplatelet therapy	1 (0.5)	0	1 (0.34)
Anticoagulation therapy	109 (51.7)	52 (58.8)	161 (54.0)
Apixaban	35 (16.6)	35 (40.2)	70 (23.5)
Rivaroxaban	22 (10.4)	3 (3.4)	25 (8.4)
Edoxaban	0	1 (1.1)	1 (0.34)
Dabigatran	7 (3.3)	3 (3.4)	10 (3.4)
Vitamin K antagonist	42 (19.9)	10 (11.5)	52 (17.4)
LMWH	3 (1.4)	0	3 (1.0)
Immunosuppressive therapy	9 (4.3)	3 (3.4)	12 (4.0)
Prednisolone	6 (2.8)	0	6 (2.0)
Methotrexate	2 (0.95)	2 (2.3)	4 (1.3)
Methotrexate + prednisolone	1 (0.47)	1 (1.1)	2 (0.67)

ARVC = arrhythmogenic right ventricular cardiomyopathy; ASA = acetylsalicylic acid; COPD = chronic obstructive pulmonary disease; HUH = Helsinki University Hospital; IQR = interquartile range; LMWH = low-molecular-weight heparin; PADIT = Prevention of Arrhythmia Device Infection Trial; SUH = Sahlgrenska University Hospital.

∗ Combined values are approximated using weighted averages of medians and IQRs from the individual cohorts.

† Estimated glomerular filtration rate of <30 mL/min.

**Table 3:** Procedure-related factors

Characteristic	HUH (n = 211)	SUH (n = 87)	Total (n = 298)
Indication for initial implantation, n (%) Primary prophylaxis	96 (45.5)	36 (41.4)	132 (44.3)
Device type, n (%)			
ICD	87 (41.2)	50 (57.5)	137 (46.0)
CRT-D	124 (58.8)	37 (42.5)	161 (54.0)
Pacemaker dependent, n (%)	49 (23.2)	21 (24.1)	70 (23.5)
Device location, n (%)			
Subcutaneous	198 (93.8)	85 (97.7)	283 (95.0)
Submuscular	13 (6.2)	2 (2.3)	15 (5.0)
Number of previous procedures, median (IQR)	1 (1–2)	1 (1–2)	1 (1–2)∗
1	134 (63.5)	57 (65.5)	191 (64.1)
2	58 (27.5)	22 (25.3)	80 (26.8)
3	19 (9.0)	8 (9.2)	27 (9.1)
Procedure duration, mean (±SD), min	26.3 (8.7)	30.8 (16.2)	27.6 (11.6)
Use of antibiotic envelope, n (%)			
All patients	123 (58.3)	30 (34.5)	153 (51.3)
High-risk patients (PADIT score ≥6)	106 (84.8)	23 (44.2)	129 (72.9)
PADIT 6p	39/49 (79.6)	5/14 (35.7)	44/63 (69.8)
PADIT 7p	45/52 (86.5)	8/19 (42.1)	53/71 (74.6)
PADIT 8p	9/10 (90.0)	2/3 (66.7)	11/13 (84.6)
PADIT 9p	7/8 (87.5)	5/11 (45.5)	12/19 (63.2)
PADIT ≥10p	6/6 (100)	3/5 (60.0)	9/11 (81.8)
Low-risk patients (PADIT score <6)	17 (19.8)	7 (20.0)	24 (19.8)
PADIT 3	0/18 (0)	0/0 (0)	0/18 (0)
PADIT 4	2/10 (20.0)	5/27 (18.5)	7/37 (18.9)
PADIT 5	15/58 (25.9)	2/8 (25.0)	17/66 (25.8)

CRT-D = cardiac resynchronization therapy defibrillator; HUH = Helsinki University Hospital; ICD = implantable cardioverter-defibrillator; IQR = interquartile range; PADIT = Prevention of Arrhythmia Device Infection Trial; SD = standard deviation; SUH = Sahlgrenska University Hospital.

∗ Combined values are approximated using weighted averages of medians and IQRs from the individual cohorts.

No major procedural differences were observed between the cohorts, except for more frequent use of an antibacterial envelope at HUH (58.3%) than SUH (34.5%). This difference was especially notable among patients with an elevated PADIT score of ≥6 (84.8% vs 44.2%). There were no preoperative pauses in the use of ASA or adenosine diphosphate blockers at SUH, whereas at HUH these agents were withdrawn 5 days before the generator replacement in 2 patients (1%).

### Procedure-related infections

3 device-related infections were documented during the follow-up: 2 cases at HUH (1 superficial infection and 1 pocket infection) and 1 case at SUH (a pocket infection), corresponding to an overall infection rate of 1.0%. The details of these patients are presented in Table 4. All patients with a device-related infection received intravenous and oral antibiotic treatment, and only 1 of them required device extraction and reimplantation. In this patient, hospitalization was substantially longer than in the other 2 cases (22 days vs 3 and 5 days).

**Table 4:** Clinical and procedural characteristics of the patients with procedure-related infections

Characteristic	Case 1	Case 2	Case 3
Hospital	HUH	HUH	SUH
Age, y	36	62	87
Sex	Female	Male	Male
PADIT score	5	6	7
Device type	ICD	ICD	CRT-D
Indication for implantation	Secondary prophylaxis	Primary prophylaxis	Primary prophylaxis
Device location	Submuscular	Subcutaneous	Submuscular
Number of previous procedures	1	3	2
Time to infection, d	10	35	188
Type of infection	Superficial	Pocket	Pocket
Antibiotic envelope use	No	Yes	Yes
Procedure duration, min	43	34	120∗
Additional clinical findings	Negative blood culture	Negative blood culture Pacemaker erosion through the wound	Negative blood culture

CRT-D = cardiac resynchronization therapy defibrillator; HUH = Helsinki University Hospital; ICD = implantable cardioverter-defibrillator; PADIT = Prevention of Arrhythmia Device Infection Trial; SUH = Sahlgrenska University Hospital.

∗ Prolonged procedure owing to relocation of the pocket from subcutaneous to submuscular.

## Discussion

This is 1 of the largest real-world studies focusing specifically on infections related to high-voltage CIED replacements. The overall infection rate of 1.0% in our cohort was low compared with previous studies reporting infection rates up to 5 times higher (see also Supplemental Table 1 in the Supplemental Appendix).^3^, ^4^, ^5^, ^6^, ^7^, ^8^, ^9^, ^10^, ^11^, ^12^, ^13^, ^14^ These findings suggest that, with proper preoperative and procedural measures, the risk of this potentially life-threatening complication is within clinically acceptable limits.

Reported infection rates in recent literature have shown considerable variation.^3^, ^4^, ^5^, ^6^, ^7^ Rates observed in the randomized trials tend to be lower than those in the epidemiologic studies, possibly owing to exclusion of high-risk patients and increased adherence to preventive measures associated with participation in a prospective trial.^3^^,^^5^^,^^9^ For example, in the PADIT,^9^ the infection rate during a 12-month follow-up period was 1.0% and 2.6% after ICD and CRT-D replacements, respectively. Another multicenter study reported an infection rate of 1.7% in a cohort of 414 high-voltage device replacements.^3^ In contrast, a Danish nationwide study covering the years 1982–2018 reported infection rates of 2.3% and 5.0% after ICD and CRT-D replacements, respectively.^5^ Our findings are in line with the randomized trials and indicate that low infection rates can also be achieved in a real-world clinical setting. This likely reflects advancements in procedural routines and preventive strategies over recent years.

According to the current guidelines, all efforts should be made to minimize device-related infections.^1^^,^^2^ The clinical workflow at the participating centers was in line with these recommendations. For instance, all procedures were performed by a senior operator under strict sterile conditions, antibiotic prophylaxis was standard practice, and no heparin or low-molecular-weight heparin bridging was used in anticoagulated patients. There were slight differences in the antibacterial prophylaxis, but these were unlikely to have had any clinically meaningful effect. The major difference between the centers concerned the use of the antibacterial envelope. The antibacterial envelope reduces the risk of device infection,^17^ but its cost-effectiveness has shown some variation in the literature.^18^, ^19^, ^20^ In general, the cost-effectiveness of the envelope improves as the absolute infection risk increases.^18^, ^19^, ^20^ Although the European Heart Rhythm Association consensus statement^1^ recommends antibacterial envelope use in high-risk patients, it does not offer specific guidance on how to identify such patients. Tools such as the PADIT^16^ and the BLISTER^19^ scores provide important means for estimating the individual infection risk. At HUH, the antibacterial envelope use is recommended for all patients with a PADIT score of ≥6 (ie, for those with an annual infection rate of >1.6%).^16^ In contrast, at SUH, the decision to use an envelope was left to the operator’s discretion. Interestingly, although the use of the antibacterial envelope was slightly higher among patients with a PADIT score of ≥6 than those with lower scores (44.2% vs 20.0%), envelope use at SUH did not increase proportionally with higher PADIT scores. In contrast, at HUH the use of the envelope showed a clear stepwise increase with higher PADIT scores. This suggests that relying solely on operator discretion may result in a more random application of the therapy rather than targeting the individuals at the highest risk, whereas a standardized protocol will help target the therapy to those most likely to benefit from the preventive measure. However, despite the institution-wide practice protocol, 1 in 6 high-risk patients (PADIT score ≥6) did not receive an antibacterial envelope at HUH, which highlights the need for continuous education and consistent protocol adherence. In contrast, the relatively common use of an antibacterial envelope in low-risk patients likely reflects the existence of additional risk factors that are not incorporated into the PADIT score,^16^ such as diabetes mellitus, chronic obstructive pulmonary disease, and heart failure.^21^

### Limitations

Despite evaluating all high-voltage generator replacements over a multiyear period at 2 large university hospitals, the overall number of procedures and especially the number of infections were limited. The small number of infections precluded any meaningful statistical comparison and in-depth analysis of additional risk factors. Future multicenter collaboration will be needed to generate more detailed data on device-related infections.

Another limitation of the study is the absence of formal statistical comparisons between the 2 centers. This was caused by restrictions related to cross-border data sharing within the European Union under the General Data Protection Regulation. Nevertheless, given the small number of events, it was evident that no clinically relevant differences existed in the infection rate between the centers and a proper statistical analysis would have been impractical.

## Conclusion

The risk of infection after high-voltage CIED replacement was 1.0% in this large cohort, indicating that infection rates under current clinical practice are within acceptable limits. Identifying the patients who may benefit the most from the use of an antibacterial envelope remains challenging. Nevertheless, our data show that favorable outcomes can be achieved by maintaining strict adherence to fundamental principles of infection prevention.

## Disclosures

J.K.: speaker honoraria and/or consultancy fees (Abbott, Biotronik, Boston Scientific, and Medtronic) and advisory board membership (Medtronic). M.S.: speaker honoraria and/or consultancy fees (Abbott, Biotronik, and Medtronic) and travel grants (Biotronik). The other authors have no conflicts of interest to disclose.

## Footnotes

Appendix Supplementary data associated with this article can be found in the online version at https://doi.org/10.1016/j.hroo.2026.01.033.

## References

[1] Blomström-Lundqvist C., Traykov V., Erba P.A. (2020). European Heart Rhythm Association (EHRA) international consensus document on how to prevent, diagnose, and treat cardiac implantable electronic device infections-endorsed by the Heart Rhythm Society (HRS), the Asia Pacific Heart Rhythm Society (APHRS), the Latin American Heart Rhythm Society (LAHRS), International Society for Cardiovascular Infectious Diseases (ISCVID) and the European Society of Clinical Microbiology and Infectious Diseases (ESCMID) in collaboration with the European Association for Cardio-Thoracic Surgery (EACTS). Europace.

[2] Baddour L.M., Esquer Garrigos Z., Rizwan Sohail M. (2024). Update on cardiovascular implantable electronic device infections and their prevention, diagnosis, and management: a scientific statement from the American Heart Association: endorsed by the International Society for cardiovascular infectious diseases. Circulation.

[3] Ellis C.R., Greenspon A.J., Andriulli J.A. (2023). Randomized trial of stand-alone use of the antimicrobial envelope in high-risk cardiac device patients. Circ Arrhythm Electrophysiol.

[4] Mittal S., Wilkoff B.L., Kennergren C. (2020). The World-wide Randomized antibiotic Envelope Infection Prevention (WRAP-IT) trial: long-term follow-up. Heart Rhythm.

[5] Olsen T., Jørgensen O.D., Nielsen J.C., Thøgersen A.M., Philbert B.T., Johansen J.B. (2019). Incidence of device-related infection in 97 750 patients: clinical data from the complete Danish device-cohort (1982–2018). Eur Heart J.

[6] Goldenberg G.R., Barsheshet A., Bishara J. (2020). Effect of fibrotic capsule debridement during generator replacement on cardiac implantable electronic device infection risk. J Interv Card Electrophysiol.

[7] Biffi M., Ammendola E., Menardi E. (2019). Real-life outcome of implantable cardioverter-defibrillator and cardiac resynchronization defibrillator replacement/upgrade in a contemporary population: observations from the multicentre DECODE registry. Europace.

[8] Uslan D.Z., Gleva M.J., Warren D.K. (2012). Cardiovascular implantable electronic device replacement infections and prevention: results from the REPLACE Registry. Pacing Clin Electrophysiol.

[9] Krahn A.D., Longtin Y., Philippon F. (2018). Prevention of arrhythmia device infection trial: the PADIT trial. J Am Coll Cardiol.

[10] Borleffs C.J., Thijssen J., de Bie M.K. (2010). Recurrent implantable cardioverter-defibrillator replacement is associated with an increasing risk of pocket-related complications. Pacing Clin Electrophysiol.

[11] Henrikson C.A., Sohail M.R., Acosta H. (2017). Antibacterial envelope is associated with low infection rates after implantable cardioverter-defibrillator and cardiac resynchronization therapy device replacement: results of the citadel and centurion studies. JACC Clin Electrophysiol.

[12] Krahn A.D., Lee D.S., Birnie D. (2011). Predictors of short-term complications after implantable cardioverter-defibrillator replacement: results from the Ontario ICD Database. Circ Arrhythm Electrophysiol.

[13] Gould P.A., Krahn A.D., Canadian Heart Rhythm Society Working Group on Device Advisories (2006). Complications associated with implantable cardioverter-defibrillator replacement in response to device advisories. JAMA.

[14] Bloom H.L., Constantin L., Dan D. (2011). Implantation success and infection in cardiovascular implantable electronic device procedures utilizing an antibacterial envelope. Pacing Clin Electrophysiol.

[15] Dai M., Cai C., Vaibhav V. (2019). Trends of cardiovascular implantable electronic device infection in 3 decades: a population-based study. JACC Clin Electrophysiol.

[16] Birnie D.H., Wang J., Alings M. (2019). Risk factors for infections involving cardiac implanted electronic devices. J Am Coll Cardiol.

[17] Tarakji K.G., Mittal S., Kennergren C. (2019). Antibacterial envelope to prevent cardiac implantable device infection. N Engl J Med.

[18] Frausing M.H.J.P., Johansen J.B., Afonso D. (2023). Cost-effectiveness of an antibacterial envelope for infection prevention in patients undergoing cardiac resynchronization therapy reoperations in Denmark. Europace.

[19] Boriani G., Kennergren C., Tarakji K.G. (2021). Cost-effectiveness analyses of an absorbable antibacterial envelope for use in patients at increased risk of cardiac implantable electronic device infection in Germany, Italy, and England. Value Health.

[20] Maclean E., Mahtani K., Honarbakhsh S. (2024). The BLISTER score: a novel, externally validated tool for predicting cardiac implantable electronic device infections, and its cost-utility implications for antimicrobial envelope use. Circ Arrhythm Electrophysiol.

[21] Polyzos K.A., Konstantelias A.A., Falagas M.E. (2015). Risk factors for cardiac implantable electronic device infection: a systematic review and meta-analysis. Europace.

