# Supplementary material for: Infections after high-voltage cardiac implantable electronic device replacements
Source: Heart Rhythm O2. 2026 Feb 18;7(5):880–6. doi: 10.1016/j.hroo.2026.01.033 (PMC13198333; doi:10.1016/j.hroo.2026.01.033)
Supplement: Supplementary Appendix [file mmc1.docx]

**Table S1. Summary of Infection Rates and Procedural Factors in ICD and CRT-D Replacements**

| **First Author and Year (Ref.)** | **Study Design** | **Sample Size (N)** | **Device** | **Type of Procedure** | **Follow-Up Duration, months** | **Infection definition** | **Antibiotic envelope** | **Infection Rate, %** |
| --- | --- | --- | --- | --- | --- | --- | --- | --- |
| Ellis et al., 2023^3^ | Prospective | 414 | PM, CRT-P, ICD, CRT-D | Generator replacement | 6 | Pocket/erosion, systemic (lead- or valve-related endocarditisor unexmplained gram-positive bacteremia) | Yes | 1.7 |
| Mittal et al., 2020^4^ | Prospective | 3371 | PM, CRT-P, ICD, CRT-D | Generator replacement, device upgrade, de novo CRT-D implantation | 21.0 (±8.3) | Superficial, pocket, systemic | Yes | 1.7 |
| Olsen et al., 2019^5^ | Retrospective | 3959 (ICD) \|  1339 (CRT-D) | ICD, CRT-D | Generator replacement | 53 (mean) | Pocket, systemic | No | 2.32 (ICD) \|  5.00 (CRT-D) |
| Goldenberg et al., 2019^6^ | Retrospective | 194 | PM, CRT-P, ICD, CRT-D | Generator replacement , device upgrade | 43.2 (mean) | Pocket, systemic | No | 2.6 |
| Biffi et al., 2019^7^ | Prospective | 983 | ICD, CRT-D | Generator change, upgrade | 12 | Pocket, systemic | No | 1.2 |
| Uslan et al., 2011^8^ | Prospective | 1031 | PM, CRT-P, ICD, CRT-D | Generator replacement | 6 | Superficial, pocket, systemic | No | 1.4 |
| Krahn et al., 2018^9^ | Prospective | 2125 (ICD) \| 831 (CRT-D) | ICD, CRT-D | Generator replacement | 12 | Pocket, systemic | No | 1.0 (ICD) \|  2.6 (CRT-D) |
| Borleffs et al., 2010^10^ | Prospective | 746 | ICD, CRT-D | Generator replacement | 38 (±32, range 0–153) | Pocket | No | 2.5 |
| Henrikson et al., 2017^11^ | Prospective | 1129 | ICD, CRT-P, CRT-D | Generator replacement, device upgrade | 12 | Superficial, pocket, systemic | Yes | 1.5 |
| Krahn et al., 2011^12^ | Prospective | 1081 | ICD, CRT-D | Generator replacement, device upgrade | 1.5 | Pocket, systemic | No | 1.7* |
| Gould et al., 2006^13^ | Retrospective | 533 | ICD | Generator replacement | 2.7 (±2.8) | Pocket, systemic | No | 1.9 |
| Bloom et al., 2010^14^ | Retrospective | 286 | ICD, CRT-D | Generator replacement, revision | 1.9 (±2.2) | Superficial, pocket, systemic | Yes | 1.0 |
| PM, pacemaker; CRT-P, cardiac resynchronization pacemaker; ICD, implantable cardioverter-defibrillator; CRT-D, cardiac resynchronization therapy defibrillator.  *no. of patients, no. of occurrences 2.1 % | | | | | | | | |
